# Walking behaviour of healthy elderly: attention should be paid

**DOI:** 10.1186/1744-9081-6-59

**Published:** 2010-10-12

**Authors:** Eling D de Bruin, André Schmidt

**Affiliations:** 1 Institute of Human Movement Sciences and Sport, ETH Zurich, HIT J 32.3; Wolfgang-Pauli-Strasse 37, CH-8093 Zürich, Switzerland

## Abstract

**Background:**

Previous studies have reported an association between executive function (EF) and measures of gait, particularly among older adults. This study examined the relationship between specific components of executive functions and the relative dual task costs of gait (DTC) in community-dwelling non-demented older adults, aged 65 years and older.

**Methods:**

Temporal (stride time, stride velocity) and spatial (stride length) gait characteristics were measured using a GAITRite^®^-System among 62 healthy community dwelling older adults while walking with and without backward counting (BC) at preferred and fast walking speeds. Specific executive functions *divided attention*, *memory *and *inhibition *were assessed using the Test for Attentional Performance (TAP). Other measures included Mini-Mental State Examination (MMSE), amount of daily medications taken, educational level and sociodemographic characteristics. Adjusted and unadjusted multivariable linear regression models were developed to assess the relations between variables.

**Results:**

High relative DTC for stride time, stride velocity and stride length were associated with divided attention at fast walking speed. High relative DTC for stride time was associated with divided attention at preferred walking speed. The association between high DTC of stride length and memory was less robust and only observable at preferred walking speed. None of the gait measures was associated with inhibition.

**Conclusions:**

Spatial and temporal dual task cost characteristics of gait are especially associated with divided attention in older adults. The results showed that the associated DTC differ by executive function and the nature of the task (preferred versus fast walking). Further research is warranted to determine whether improvement in divided attention translates to better performance on selected complex walking tasks.

## Background

In the growing population of older people falling is a common problem. Approximately 30% of older adults experience a fall each year [1-3], and fall incidence is even higher (50%) in women aged 85 and above [4]. Gait problems and weakness are a common specific precipitating cause for falls [5], and persons with a walking disability have an increased risk of repeated falls [6] and a reduced survival compared to peers with normal walking [7,8]. In light of these negative consequences, much research has been directed towards the determinants of walking disability. There are indications that the influence of motor and sensory impairments on falls is in part moderated by executive functioning [9].

Various studies have shown that, in contrast to past believes, gait performance is not only an automated sequence of body movements. Cognitive functions also play an important role in the control of gait. These cognitive functions are mostly attributed to so-called executive control processes of the human brain [10-12]. A recent review on this topic summarizes the interplay between executive functions, attention and gait [13]. Executive function (EF) refers to cognitive processes that control and integrate other cognitive activities [14,15], and this term has been used to describe a group of cognitive actions that include: dealing with novelty, planning and implementing strategies for performance, monitoring performance, using feedback to adjust future responding, vigilance, and inhibiting task-irrelevant information [14] of lower level, more modular, or automatic functions [16]. Common tasks of daily life require attention, rapid motor planning process, and effective inhibition of irrelevant or inappropriate details. Older adults, however, experience increasing difficulties in maintaining multiple task rules in working memory [17].

Existing knowledge about the interplay between EF and gait is mostly derived from studies that measured and reported EF as a composite score [12,18,19]. Relatively few studies have focused on the age-related deficits in specific components of executive function and most of these studies were based on a traditional set of tests of executive function, without detailing specific components. The conclusions drawn from these studies might, therefore, be limited by their methodologies. The putative executive measures might not load on a single executive construct, and might overlap with each other [20,21]. The differential breakdown for the executive functioning performance across patients with chronic schizophrenia, for example, suggests that the fractionation of central executive functioning occurs in schizophrenia and not all EF components are in each case equally impaired in their performance [22]. Preliminary evidence suggests that also in normal aging there are selective deficits in executive function rather than a general decline [23-26]. These studies, thus, suggest that the fractionation of executive function is necessary when the EF effects on gait are studied.

Three components of EF (EF_comp_) are mentioned in the literature as being related to gait: "working memory"[27], "divided attention"[11], and "inhibition"[28]. It is unclear whether these components relate to gait with comparable portions. Furthermore, it is unclear whether these measures are independent in explaining variability in gait. The aim of this study was to determine whether dual-task costs of gait in healthy elderly are explained by these three EF_comp_. We hypothesised that *divided attention*, *memory *and *inhibition *each explain comparable portions of dual-task costs of gait measures in elderly community dwellers.

## Methods

The sample for this cross-sectional analysis consisted of sixty-nine healthy elderly subjects who consented to perform the measurements. Subjects were free of any orthopaedic disorder of the lower limb that might affect their gait, and did not report acute pain or any other complaint likely to influence walking. Subjects were recruited from the local community using various strategies. Senior subjects attending exercise classes of the Academic Sports Club Zurich (ASVZ "Akademischer Sportverein Zürich") were approached shortly before their training started. The study was also presented at a "senior university" lecture at the University of Zurich, Zurich, Switzerland. After a short presentation leaflets about the study were distributed in which interested individuals were encouraged to contact us. Furthermore, sport events for seniors were visited to recruit volunteers. In addition an advertisement in the ETH magazine "ETH Life Print" with information on the study was published (edition May, 2008). The institutional review board of the ETH Zurich provided approval for the project and all subjects provided written informed consent.

Inclusion criteria were age 65 or above and the ability to walk without walking aids. Exclusion criteria were a score below 25 on the Mini Mental Status Examination (MMSE)[29], medically diagnosed gait impairments of neurologic and/or orthopaedic origin, and any musculoskeletal impairments that influence gait pattern.

### Gait analysis

In order to assess temporal-spatial characteristics of gait we used the GAITRite^® ^system (CIR Systems Inc., Havertown (PA), USA). The system was 13 m long and 0.89 m wide, with an active sensor area of 7.32 m long and 0.61 m wide. The sampling rate of the system is 80 Hz. Spatial-temporal gait parameters were processed and stored using the application software. Studies have reported both high reliability and validity of the GAITRite^® ^system for measuring spatial and temporal gait characteristics in older subjects [30-34].

### Procedure

Testing was performed in the gait laboratory of the City Hospital Waid in Zurich, Switzerland. To measure steady state walking, the central 7.32 m active sensor area of the GAITRite^® ^system was used as the test distance. During the measurements, the subjects walked on the walkway while wearing their own comfortable clothing and low-heeled habitual shoes. Since mean values of eight strides have been shown to be appropriately representing gait characteristics and can be considered as representative of normal gait[33] we ensured the capturing of at least 25 steps per test condition. Each subject was instructed to walk the walkway three times, in randomised order, at I) self-selected comfortable speed, II) a self-selected higher speed, III) a self-selected comfortable speed with a working memory task, and IV) a self-selected higher speed with a working memory task, making a total of 12 walks per individual. Participants were not specifically instructed to prioritize either one of both tasks, but were asked to combine both tasks at their best capacity.

In the working memory task the subjects walked while reciting out loud serial subtractions of seven, starting from a given random number between 200 and 250. Before performing the task while walking, the participants were allowed to practice while sitting, in order to evaluate basic problems in calculating. The sequence of numbers was reported on protocols. Evaluation of performance on the serial 7 subtraction included the total number of subtractions and the number of mistakes made during calculation. Only successful trials were used for further data analysis.

Since the relation between measures of gait and executive function were demonstrated for complex conditions [35,36], and we wanted to account for individual differences [13] we examined the relation for the interference between task conditions I vs. III, and II vs. IV. This was expressed as relative dual task costs (DTC) of walking with the formula DTC [%] = 100 * (single-task score - dual-task score)/single-task score [37]. Stride length (cm), stride velocity (cm/s) and stride time (s) of the left foot were evaluated. Values were expressed as Mean ± SD.

### Cognitive assessment

All subjects underwent neuropsychological assessment with the Test for Attentional Performance ("*Testbatterie zur Aufmerksamkeitsprüfung" *(TAP))[38], which took less than 20 minutes, across the cognitive domains attention, memory and executive functions. The core of the procedures is reaction time tasks of low complexity allowing the evaluation of very specific deficiencies. The tasks consist of simple and easily distinguishable stimuli that the participants react to by a simple motor response. The procedures included in the test battery were: Divided Attention, Go/No-Go, and Working Memory. The TAP test has previously been shown to be both reliable and valid [39,40] and offers values for normative and brain injured populations [41,42].

• The divided attention performance assessment is realized by a visual and an acoustical task. The visual task consists of crosses that appear in a random configuration in a 4 × 4 matrix. The subject has to detect whether the crosses form the corners of a square. The acoustical task includes a regular sequence of high and low beeps. The subject has to detect an irregularity in the sequence.

• In the go/no-go tasks, the subject has to react selectively to one class of stimuli but not to others. For testing a go/no-go-task was realized with a high memory load with five stimuli, squares with different textures, where two were targets. The aim of this examination is an assessment of the capacity of focused attention (reject irrelevant information).

• The working memory task requires a continuous control of the information flow through short-term memory. For this, numbers are presented on the screen that must be compared with previously exposed numbers. The repetition of a number within a short interval has to be answered by pressing a key.

For these three tests of EF_comp_, we analysed the number of omissions for the subtests "divided attention" and "working memory". In the subtest "Go/No-Go" the number of errors was taken. Each of these tests took 5 minutes. The subject had the opportunity to practice by means of a few examples and was allowed to ask the instructor for help. During the main test the subjects had to act on their own.

### Sociodemographic characteristics and other measures

Sociodemographic variables and descriptive variables were obtained through the use of a standardized questionnaire and by a semi-structured interview. These variables included age, gender, body height and weight, years of completed education, living status, self-reported chronic conditions, use of medication, falls in the previous half year, and concern about falling [43] (Table 1).

**Table 1:** Baseline and gait characteristics of subjects

	Subjects
Age, years (mean ± SD)	72.5 ± 5.9

Weight (kg)	70.2 ± 10.7

Height (cm)	170 ± 7.9

Females (N, %)	28, 44%

MMSE (points)	28.1 ± 1.3

FES-I (points)	17.3 ± 1.5

Education (years)	14.6 ± 2.6

Living status	
One person household (%)	31
Multiple persons household (%)	69

Number of self-reported chronic diseases (%)	
Joint pain	27.4
Hypertension	29.0
none	35.5

Use of hearing aids (%)	19.4

Use of visual aids (%)	37.1

Stride velocity (CS; mean ± SD; single task/dual task; m/s)	1.32 ± 0.17/1.13 ± 0.3

Stride time (CS; mean ± SD; single task/dual task; seconds)	1.1 ± 0.1/1.3 ± 0.7

Stride length (CS; mean ± SD; single task/dual task; m)	1.43 ± 0.15/1.35 ± 0.18

Stride velocity (HS; mean ± SD; single task/dual task; m/s)	1.74 ± 0.2/1.28 ± 0.34

Stride time (HS; mean ± SD; single task/dual task; seconds)	0.9 ± 0.1/1.2 ± 0.7

Stride length (HS; mean ± SD; single task/dual task; m)	1.62 ± 0.16/1.41 ± 0.19

MMSE: mini mental state examination; FES-I: falls efficacy scale-international; CS: self-selected comfortable speed; HS: self-selected higher speed

### Data analysis

Descriptive statistics were used to evaluate participants' demographic characteristics (Table 1). Changes in DTC between test conditions (preferred and fast walking) were compared with the paired *t*-test.

A series of simultaneous linear regression models, both unadjusted and adjusted for age, cognition (MMSE), medication use, and years of education, were constructed to examine the association of the main outcome (relative DTC of walking) with the EF_comp_. The contribution of each variable to the regression equation was described in terms of the beta coefficient and the corresponding statistical significance. The adjusted multivariate coefficient of determination (*R*^2^) was used to describe the variability in relative DTC of walking explained by all of the variables of interest entered into the model.

To ensure that the reliability of the regression was not compromised by multicollinearity, we determined the tolerance of each variable before entry into the equation. The experiment-wise Type I error rate was set at 0.05 for the statistical tests. All statistics were performed with SPSS 17.0.

## Results

### Subjects

Seven participants had to be excluded from the analysis. Four individuals were not able to combine the walking test with the serial subtraction task. These individuals were only able to perform both tasks independently. Even after adjusting the task to counting backwards in ones these individuals were not able to combine the tasks. One individual had a MMSE score of 23 and two individuals withdrew because they were ill at the scheduled measurement day. The resulting group consisted of 34 men and 28 women, with a mean age of 72.5 ± 5.9 years (range: 65 - 85 years). Demographic and gait measurement related characteristics of the subjects are presented in table 1.

### Linear regression analysis

A high tolerance and a variance inflation factor (VIF) indicated that the reliability of the estimate of the regression coefficient was not significantly affected by collinearity between the independent variables in the respective equations.

Table 2 reports the results of the adjusted analysis for the DTC of stride velocity. The *R*^2 ^= .05 for preferred walking speed Step 1, the change (Δ) *R*^2 ^= .11 for preferred walking speed Step 2 (*p *= .086). The *R*^2 ^= .38 for fast walking speed Step 1, and Δ*R*^2 ^= .27 for fast walking speed Step 2 (*p *< .001).

**Table 2:** Regression model for Stride Velocity

	*B*	*SE B*	β
**Preferred walking speed**			
			
**Step 1**			
Constant	-81.72	75.02	
Age	0.57	0.49	.16
MMSE	1.54	2.3	.09
Medications	0.94	1.88	.07
Years of education	0.71	1.07	.09

**Step 2**			
Constant	-74.99	75.02	.16
Age	0.10	0.54	.003
MMSE	2.01	2.30	.12
Medications	1.49	1.91	.11
Years of education	1.43	1.10	.18
Divided attention	1.24	0.98	.17
Memory	1.45	0.80	.28
Inhibition	0.20	0.50	.05

			

**Fast walking speed**			
			
**Step 1**			
Constant	111.36	60.73	
Age	0.43	0.40	.14
MMSE	-4.53	1.86	-.32*
Medications	0.37	1.52	.03
Years of education	0.70	0.86	.10

**Step 2**			
Constant	129.87	54.28	
Age	-0.004	0.39	-.001
MMSE	-4.59	1.64	-.33**
Medications	0.31	1.36	.03
Years of education	1.08	0.78	.16
Divided attention	3.38	0.70	.54***
Memory	0.004	0.57	.001
Inhibition	-0.18	0.36	-.06

MMSE: mini mental state examination

Note: *R*^2 ^= .05 for preferred walking speed Step 1, Δ*R*^2 ^= .11 for preferred walking speed Step 2 (*p *= .086); *R*^2 ^= .38 for fast walking speed Step 1, Δ*R*^2 ^= .27 for fast walking speed Step 2 (*p *< .001).

* < .05; ** < .01; ***< .001

The adjusted analysis for the DTC of stride time and stride length are presented in Tables 3 and 4. *R*^2 ^= .04 for preferred walking speed Step 1, Δ*R*^2 ^= .20 for preferred walking speed Step 2 (*p *< .01); *R*^2 ^= .22 for fast walking speed Step 1, Δ*R*^2 ^= .28 for fast walking speed Step 2 (*p *< .001). *R*^2 ^= .04 for preferred walking speed Step 1, Δ*R*^2 ^= .14 for preferred walking speed Step 2 (*p *< .05); *R*^2 ^= .21 for fast walking speed Step 1, Δ*R*^2 ^= .12 for fast walking speed Step 2 (*p *< .05).

**Table 3:** Regression model for Stride time

	*B*	*SE B*	β
**Preferred walking speed**			
			
**Step 1**			
Constant	-78.05	181.04	
Age	0.51	1.18	.06
MMSE	0.42	5.56	.01
Medications	-0.88	4.53	-.03
Years of education	3.44	2.58	.18

**Step 2**			
Constant	-43.34	173.55	
Age	-0.67	1.24	-.08
MMSE	0.73	5.24	.02
Medications	-0.91	4.36	-.03
Years of education	4.55	2.50	.24
Divided attention	8.01	2.24	.46***
Memory	0.64	1.83	.05
Inhibition	-0.09	1.14	-.01

			

**Fast walking speed**			
			
**Step 1**			
Constant	198.88	260.24	
Age	0.49	1.70	.04
MMSE	-9.79	7.99	-.17
Medications	-1.64	6.52	-.04
Years of education	5.08	3.70	.18

**Step 2**			
Constant	271.80	235.19	
Age	-0.84	1.68	-.07
MMSE	-10.53	7.11	-.18
Medications	-2.71	5.90	-.06
Years of education	5.94	3.39	.22
Divided attention	14.44	3.04	.57***
Memory	-1.82	2.48	-.10
Inhibition	-0.95	1.55	-.07

MMSE: mini mental state examination

Note: *R*^2 ^= .04 for preferred walking speed Step 1, Δ*R*^2 ^= .20 for preferred walking speed Step 2 (*p *< .01); *R*^2 ^= .22 for fast walking speed Step 1, Δ*R*^2 ^= .28 for fast walking speed Step 2 (*p *< .001).

* < .05; ** < .01; *** < .001

**Table 4:** Regression model for Stride length

	*B*	*SE B*	β
**Preferred walking speed**			
			
**Step 1**			
Constant	-26.44	32.99	
Age	0.12	0.22	.08
MMSE	0.67	1.01	.09
Medications	0.79	0.83	.14
Years of education	0.23	0.47	.07

**Step 2**			
Constant	-23.51	32.82	
Age	-0.08	0.23	-.06
MMSE	0.84	0.99	.12
Medications	1.30	0.82	.22
Years of education	0.58	0.47	.17
Divided attention	-0.67	0.42	-.21
Memory	0.98	0.35	.43**
Inhibition	0.05	0.22	.03

**Fast walking speed**			
			
**Step 1**			
Constant	60.05	29.23	
Age	0.20	0.19	.13
MMSE	-2.47	0.90	-.35**
Medications	0.78	0.73	.14
Years of education	0.47	0.42	.14
**Step 2**			
Constant	65.41	29.15	
Age	0.01	0.21	.01
MMSE	-2.42	0.88	-.34**
Medications	0.84	0.73	.15
Years of education	0.66	0.42	.20
Divided attention	1.02	0.38	.33**
Memory	0.20	0.31	.09
Inhibition	-0.01	0.19	-.01

MMSE: mini mental state examination

Note: *R*^2 ^= .04 for preferred walking speed Step 1, Δ*R*^2 ^= .14 for preferred walking speed Step 2 (*p *< .05); *R*^2 ^= .21 for fast walking speed Step 1, Δ*R*^2 ^= .12 for fast walking speed Step 2 (*p *< .05).

* < .05; ** < .01; *** < .001

Results for the unadjusted analysis (Table 5) show *R*^2 ^= .10 for DTC stride velocity at preferred walking speed (*p *= .097), *R*^2 ^= .18 for DTC stride time at preferred walking speed (*p *< .01), *R*^2 ^= .11 for DTC stride length at preferred walking speed (*p *= .079); *R*^2 ^= .31 for DTC stride velocity at fast walking speed (*p *< .001), *R*^2 ^= .28 for DTC stride time at fast walking speed (*p *< .001), and *R*^2 ^= .17 for DTC stride length at fast walking speed (*p *< .05).

**Table 5:** Results of the unadjusted multiple regression models for stride velocity, time, and length at preferred and fast walking speeds.

	*B*	*SE B*	β
**Preferred walking speed**			
			
**Stride velocity**			
Constant	6.32	4.23	
Divided attention	1.32	0.96	.18
Memory	1.06	0.70	.20
Inhibition	0.20	0.47	.05

**Stride time**			
Constant	-0.91	9.68	
Divided attention	7.66	2.21	.44***
Memory	-0.56	1.59	-.04
Inhibition	-0.04	1.07	-.01

**Stride length**			
Constant	4.74	1.84	
Divided attention	-0.62	0.42	-.19
Memory	0.76	0.30	.33*
Inhibition	0.10	0.20	.06

**Fast walking speed**			
			
**Stride velocity**			
Constant	15.87	3.16	
Divided attention	3.47	0.72	.56***
Memory	-0.04	0.52	-.01
Inhibition	0.11	0.35	.03

**Stride time**			
Constant	1.17	13.16	
Divided attention	14.07	3.00	.56***
Memory	-2.89	2.16	-.16
Inhibition	-0.34	1.45	-.03

**Stride length**			
Constant	8.35	1.73	
Divided attention	1.12	0.39	.36**
Memory	0.14	0.28	.06
Inhibition	0.18	0.19	.11

Note: *R*^2 ^= .10 for DTC stride velocity at preferred walking speed (*p *= .097), *R*^2 ^= .18 for DTC stride time at preferred walking speed (*p *< .01), *R*^2 ^= .11 for DTC stride length at preferred walking speed (*p *= .079); *R*^2 ^= .31 for DTC stride velocity at fast walking speed (*p *< .001), *R*^2 ^= .28 for DTC stride time at fast walking speed (*p *< .001), and *R*^2 ^= .17 for DTC stride length at fast walking speed (*p *< .05).

* < .05; ** < .01; *** < .001

The mean relative DTC at preferred walking speed was 14.7 ± 20.7% (stride velocity), 19.7 ± 49.5% (stride time), and 5.9 ± 9% (stride length). The mean relative DTC at fast walking speed was 26.4 ± 17.6% (stride velocity), 31.4 ± 71.6% (stride time), and 12.8 ± 8.8% (stride length). The change in DTC caused by the difference in walking speed during testing was significant for all dependent variables: stride velocity, *p *< .001; stride time, *p *< .01; stride length, *p *< .001.

The regression analysis showed that the relative DTC at preferred walking speed was explained by divided attention (stride time, tables 3 &5), and working memory (stride length, tables 4 &5).

The regression analysis showed that the relative DTC at fast walking speed was explained by the MMSE & divided attention (stride velocity, tables 2 &5), divided attention (stride time, tables 3 &5), and MMSE & divided attention (stride length, tables 4 &5).

## Discussion

The aim of this study was to determine to which degree the relative dual task costs of walking in healthy elderly are explained by three EF_comp_. We hypothesised that *divided attention*, *memory *and *inhibition *would each explain comparable portions of gait measures in elderly community dwellers. To our knowledge, this is the first investigation that took specific executive functions to study the relation with gait with a dual-task assessment design.

Previous work from other groups already provided evidence that changes in gait during dual tasking are mediated by reduced executive functioning [18]. In our cohort of older adults, individual gait characteristics were especially associated with the specific executive function *divided attention *and to a lesser extend with *memory*. These distinct association patterns remained independent of age, health status, global cognition and the level of education. The results of this study show that even in healthy subjects especially divided attention is required for gait and this suggests that changes in gait in community dwelling older individuals might indeed not only be caused by changes in more peripheral systems, however, these seem to be due to selective changes in functional properties of the brain, e.g. divided attention. Divided attention, however, is a complex construct. The consequences of limitations in divided attention are known to be profound and, if persistent, rapidly escalate into comprehensive cognitive impairments [44]. This study shows that divided attention also affects walking behavior.

It should be noted that the group of persons that we investigated primarily consisted of active, fit older adults from a higher socio-economic background. This limits the possibility to generalize our findings to the older population at large. The relationships between measures of cognitive functioning and gait can be expected to be more pronounced when a group of older people with more variation in physical functioning is investigated since, as previous research suggested, this physical functioning is related to executive functioning [35,45,46]. In addition, it can be speculated that different relations might emerge when specific groups of older adults are investigated, e.g. demented Parkinson's disease (PD) patients and Alzheimer's disease (AD) patients [47]. These arguments indicate that caution in generalizing our results to older adults in general is indicated.

The results showed that the relative dual task costs of walking relate differently to specific executive functions. Further research is needed to determine whether executive function abilities also causally relate to walking in elderly. It can be hypothesized from this study that interventions that want to influence dual task costs of gait should especially focus on divided attention.

## Limitations

A limitation of this study was that we did not directly measure different regions and networks of the brain, that were previously reported to be associated with gait measures, with more advanced techniques, e.g., brain magnetic resonance imaging [48]. We rather assessed global neuropsychological performance. Therefore, we can only speculate about the specific brain regions involved underlying the neuropsychological test that we used. Previous work has shown that spatial and temporal characteristics of gait are both associated with distinct brain networks in older adults and reported that no gait measures were associated with, amongst others, regions of the memory domains [48]. Our results seem to be, therefore, somewhat at variance with these results since we found some association between the EF_comp _memory and stride length at preferred walking speed. However, it might well be that the use of another type of neuropsychological assessment would have resulted in differing findings since different types of assessment that pretend to measure the same cognitive construct do not relate to each other and are rather complementary to each other [49].

A large proportion of the variance in DTC of gait is left unexplained in our study. We are aware that many other factors may contribute to the variance in relative DTC of gait, such as genetic and environmental factors [50], interactions between sensory/sensorimotor and cognitive functions [51], or visual observation skills [52]. The fact that we did not include these parameters in our study could be regarded as another limitation of this study. However, we were not aiming to find and explain as many factors as possible that contribute to the total variance of relative DTC of walking in a cohort of elderly. Our aim was to assess the specific contributions to this variance of three EF_comp_. This study showed that especially divided attention contributes to the variance, which has potential relevance for future intervention studies.

## Conclusions

Spatial and temporal dual task cost characteristics of gait are especially associated with divided attention in older adults. The results showed that the associated DTC differ by executive function and the nature of the task (preferred versus fast walking). Further research is needed to determine whether improvement in divided attention translates to better performance on selected complex walking tasks. The findings of this study of walking characteristics of well-functioning older adults prepare the groundwork for future interventional type studies to examine causality between DTC of walking and improvements in divided attention.

## Competing interests

The authors declare that they have no competing interests.

## Authors' contributions

EDB, the guarantor, initiated the study, participated in its design, monitored progression and decided on the analytical strategy. He drafted the final manuscript and critically revised the manuscript for its content. AS conceived of the study, carried out the study, and drafted the first version of the manuscript. Both authors read and approved the final manuscript.
